# Double Muscular Thenar Branch and Anatomical Variation of Thenar Muscle Innervation Observed in a Cadaver

**DOI:** 10.7759/cureus.5859

**Published:** 2019-10-07

**Authors:** Konstantinos Natsis, Maria Tzika, George K Paraskevas, Marinos Karanassos, Nikolaos Lazaridis

**Affiliations:** 1 Department of Anatomy and Surgical Anatomy, Aristotle University of Thessaloniki, Thessaloniki, GRC; 2 Department of Orthopaedics, Aristotle University of Thessaloniki, Thessaloniki, GRC

**Keywords:** recurrent nerve, loop, first common palmar digital nerve, origin, accessory branch

## Abstract

The muscular thenar branch typically arises from the median nerve distal to the transverse carpal ligament and supplies the thenar musculature. In the present cadaveric case, the existence of double muscular thenar branches is described. The two nerves originated from the first common palmar digital nerve and gave off five terminal muscular branches. The proximal nerve supplied the abductor pollicis brevis (two branches) and the opponens pollicis (two branches) muscles, whereas the distal muscular thenar branch presented a loop and innervated the superficial head of flexor pollicis brevis muscle. Variations of the muscular thenar branch in the anatomical and surgical literature are discussed, along with the potential implications during surgical treatment of carpal tunnel syndrome (CTS).

## Introduction

Thumb is responsible for the complex function of the hand. The muscular thenar branch (MTB) of the median nerve (MN), also known as recurrent or motor nerve branch, typically arises distal to the transverse carpal ligament (TCL); it recurs around the distal border of the TCL and enters the abductor pollicis brevis (APB), opponens pollicis (OP), and the superficial head of the flexor pollicis brevis (FPB) muscle [1]. Variations of the origin, course, and the number of MTB are common, and typical anatomy is reported in less than 75% of cases studied in the literature [2-5]. Thus, knowledge of the MTB anatomy and topography is essential for clinical practice.

MN motor fibers are usually affected in carpal tunnel syndrome (CTS), the most common compression mononeuropathy. Patients present with thenar atrophy and sensory deficit and alterations over the MN distribution area. During the surgical release, iatrogenic injury to the MTB is the most disastrous complication that may occur, as it spares motor control of the thumb postoperatively. Thus, it has been called the “million-dollar injury”, due to the high malpractice compensation for the disability caused [6]. In the current study, an atypical branching pattern of MN with double MTB is described comprehensively.

## Case presentation

During routine dissection of a 70-year-old formalin-fixed male cadaver, at the Department of Anatomy and Surgical Anatomy, Aristotle University of Thessaloniki, an atypical branching pattern of the right MN was observed. By means of the classical method of anatomic dissection, after removal of the skin and subcutaneous tissue of the wrist and hand area, MN was identified proximal to the TCL and afterwards, TCL was divided.

**Figure 1:**
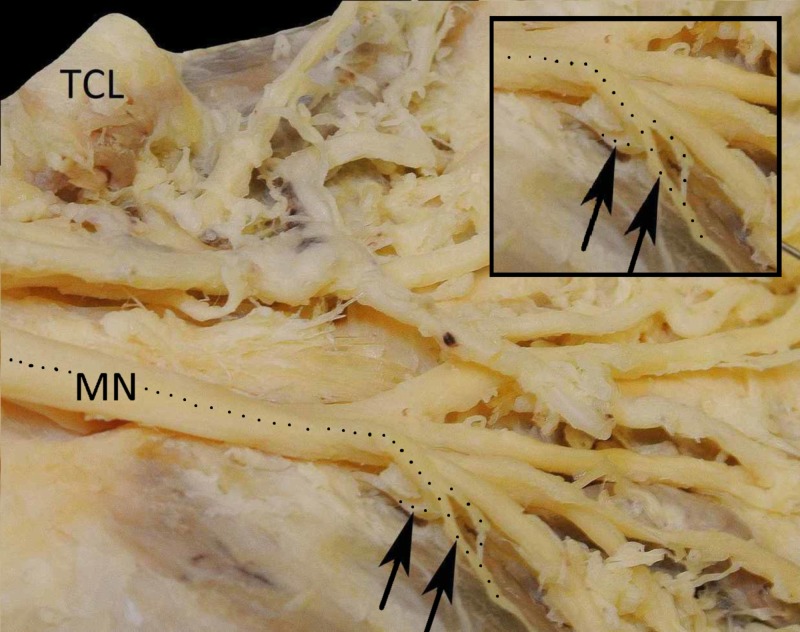
The origin of motor thenar branches The MN is divided into a medial and a lateral branch after it passes underneath the TCL. The lateral branch gives off the first common palmar digital nerve for the thumb and the radial side of the index. The TCL is dissected and the origin of the two motor thenar branches (arrowheads) is presented. The course of motor thenar branches is shown by a dotted line, while the loop of the distal motor thenar branch is emphasized. MN, median nerve; TCL, transverse carpal ligament

The branches of the MN were identified and two MTBs were found arising from the first common palmar digital nerve. The proximal branch was thicker and originated laterally, distal to the TCL (Figure 1).

**Figure 2:**
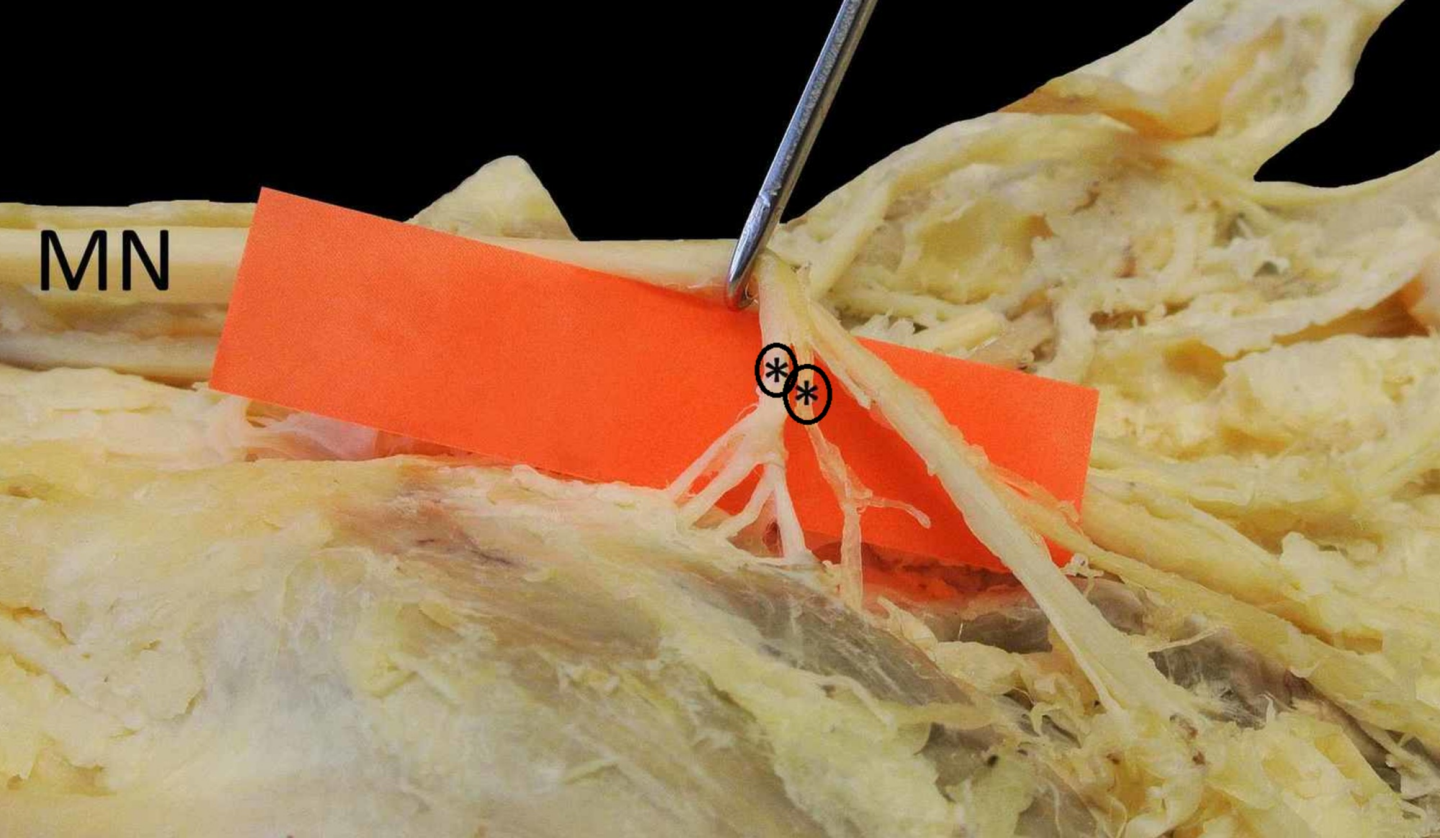
Terminal muscular branches After further dissection, the five terminal branches of the double motor thenar branches (*), arising from MN, are observed, inserting into the musculature of the thenar. Due to traction, the nerve loop of the distal motor thenar branch was divided. MN, median nerve

Then, it curved off and travelled proximally in order to divide into four motor branches directed to the thenar muscles (Figure 2).

The second MTB also originated laterally from the first common palmar digital nerve and it presented a loop, which was accidentally dissected during further dissection. These extraligamentous branches were traced to the musculature as they passed through the thenar fascia and inserted into the thenar muscles independently. The first MTB was responsible for the innervation of APB (two branches) and the OP (also two branches), while the second MTB supplied the superficial head of FPB muscle.

The cause of death was not related to the current study. There was no demonstrable pathology or evidence of previous surgical procedures undertaken in the region of interest, while no other abnormalities were detected in the ipsilateral upper limb.

## Discussion

Typically, MN travels posterior to the TCL and it splits into a lateral and a medial nerve branch. The lateral terminal branch gives off the MTB for the innervation of the thenar musculature and the first common palmar digital nerve, while the medial branches off the second and third common palmar digital nerves. The MTB presents a recurrent curvature at its origin site, as it turns proximally, lying over the FPB muscle before its division to its terminal muscular branches [1]. Many variations have been reported in the anatomical and surgical literature, concerning MTB’s origin site, course, and branching pattern. 

The classification of MTB’s origin site as extraligamentous, subligamentous, transligamentous, and more recently preligamentous, in relation to the TCL has been widely studied and there is great differentiation concerning the incidence of each subtype [4,7]. In the study of Lanz, MN was studied during carpal tunnel exploration and it was classified into four types. Type I (88.2%) includes cases of MTB’s origin from the first common palmar digital nerve and variant topography (extraligamentous, subligamentous, and transligamentous MTB). Type II includes cases of accessory MN branches originating distal to the carpal tunnel (7.2%), whereas cases of preligamentous MN division (type III) and accessory MN branches originating proximal to the carpal tunnel (type IV) were found in 4.6%. Origin of a single MTB from the first common palmar digital nerve distal to the TCL is reported in 46% of the cases that were included in type I (type Ia) [7]. In our case, two true MTBs were observed, arising from the first common palmar digital nerve distal to the TCL, while five terminal muscular braches were detected.

Extraligamentous accessory MTBs that supply the superficial belly of FPB muscle have been reported in two out of 10 cases in the work of Falconer and Spinner, whereas in all of these cases, the main MTB was transligamentous. Furthermore, in the same study, MTB originated from the MN trunk in all cases, while bifurcation of the lateral branch of MN into the common palmar digital nerve for the thumb and the radial digital nerve for the index was noted in only one case [8]. Various cadaveric case reports have also been published, describing the presence of multiple MTBs. Innervation of the thenar musculature by seven MTBs, arising from the MN trunk and proper palmar digital nerve to the lateral side of the thumb, multiple accessory MTBs originating proximally to the TCL or from the second common palmar digital nerve have been documented, indicating the variability of MTB’s anatomy [9-11].

In the comprehensive research of Amadio on patients undergoing carpal tunnel release due to CTS, variations in the anatomy of MTB were found in 25.1% of the total cases, while in 2.9% of the cases, two MTBs were noticed [2]. In another series of MN decompression, Al-Qattan et al. found that in 56% of their specimens, one extraligamentous MTB was present, while in the subligamentous MTB cases (34%), one case of double MTB was detected. Moreover, 46% of the extraligamentous branch cases and all preligamentous and transligamentous (9%) cases were associated with a hypertrophic muscle over the TCL [4].

As far as the MTB’s branching pattern is concerned, Mumford et al. studied MTB’s anatomy, reporting that only 45% of the MTB presented typical trifurcation into muscular branches for APB, OP, and FPB. The authors also mentioned that in 75%, an accessory MTB was found and it supplied the FPB, as it occurs in our case. Additionally, the accessory MTB arose from the first common palmar digital nerve in five cases [12]. Olave et al. after dissecting 60 cadaveric palms, observed three distinct distribution patterns. Type I (50%) included typical three-pattern branching to APB, OP, and FPB muscles, in type II (40%), MTB provided supply only to APB and OP muscles, while in type III (10%), the MTB branches originated from a common trunk before the thenar fascia [13]. Innervation of OP by two muscular branches of MTB was found in 10% to 26.7% of cases [12-13]. In the current study, the first MTB innervated APB and OP (type II in Olave’s classification system), whereas the superficial head of FPB was supplied by the second MTB. Both MTBs originated radially from the first common palmar digital nerve.

In another study, by Alp et al., including 144 cadaveric hands, only in 16% of the total cases the MTB branched off before entering the thenar fascia, as it occurred in our case. In 84% of the cases (group I), there was a main MTB arising from the MN, while the three-branching pattern to APB, OP, and FPB was found in 47.9% (subtype IA). In 7.6% of the total specimens, an accessory MTB was observed arising from the first common palmar digital nerve and innervating the FPB muscle (subtype IC), while in 0.7% the FPB muscle received dual innervation by the main and the accessory MTB. In group II (13.2%), the authors included all cases in which the thenar muscles were supplied by two nerve branches present before the thenar fascia, including one case where an accessory MTB was found originating from the first common palmar digital nerve and innervating the FPB along with the main MTB (subtype IIAa). In 8.2% of the whole specimens, the accessory MTB was responsible for the innervation of FPB muscle. Groups III (2.1%) and IV (0.7%) included cases of three and four MTBs, respectively [14].

Variations of MTB’s anatomy could affect the outcome of CTS surgery. Compression of the MTB causes thenar muscle atrophy and it may occur due to CTS or independently. Electrophysiologic findings are essential in the diagnosis; however, findings that indicate selective MTB compression may be attributed to CTS, as the MN motor fibers are located right beneath the TCL in cases of MTB’s typical extraligamentous origin and may be affected first [15]. In an extended survey of the British Society for Surgery of the Hand, regarding the risk of MTB’s injury during CTS surgery, 71% of the participants responded that there is no need for total exploration in order to preserve the MTB, as the “million dollar injury” is rather rare [16]. However, “normal anatomy” was found in less than 75% of the cases studied in the referred literature [2-5]. Therefore, thorough knowledge of the MTB’s anatomy is essential for surgeons, to succeed in the total MN release and symptoms’ relief, without jeopardizing further nerve damage.

## Conclusions

Anatomical variations of MTB, including atypical origin, number, topography, and terminal MTB branching pattern, may alter the symptomatology of CTS patients and complicate the surgical procedure. In the presented case, two MTBs were found arising from the first common palmar digital nerve and giving off five terminal muscular branches in total, while one of them presented a loop. This case report aims in highlighting the significance of awareness and knowledge of MTB’s variant anatomy during CTS surgery, to avoid nerve damage and postoperative complications.
